# Assessment and Evaluation of Different Machine Learning Algorithms for Predicting Student Performance

**DOI:** 10.1155/2022/4151487

**Published:** 2022-05-09

**Authors:** Yazan A. Alsariera, Yahia Baashar, Gamal Alkawsi, Abdulsalam Mustafa, Ammar Ahmed Alkahtani, Nor'ashikin Ali

**Affiliations:** ^1^Department of Computer Science, College of Science, Northern Border University, Arar, Saudi Arabia; ^2^Faculty of Computing and Informatics, Universiti Malaysia Sabah (UMS), Labuan, Malaysia; ^3^Faculty of Computer Science and Information Systems, Thamar University, Yemen; ^4^College of Graduate Studies, Universiti Tenaga Nasional, Kajang 43000, Malaysia; ^5^Institute of Sustainable Energy (ISE), Universiti Tenaga Nasional, Kajang 43000, Malaysia

## Abstract

Student performance is crucial to the success of tertiary institutions. Especially, academic achievement is one of the metrics used in rating top-quality universities. Despite the large volume of educational data, accurately predicting student performance becomes more challenging. The main reason for this is the limited research in various machine learning (ML) approaches. Accordingly, educators need to explore effective tools for modelling and assessing student performance while recognizing weaknesses to improve educational outcomes. The existing ML approaches and key features for predicting student performance were investigated in this work. Related studies published between 2015 and 2021 were identified through a systematic search of various online databases. Thirty-nine studies were selected and evaluated. The results showed that six ML models were mainly used: decision tree (DT), artificial neural networks (ANNs), support vector machine (SVM), K-nearest neighbor (KNN), linear regression (LinR), and Naive Bayes (NB). Our results also indicated that ANN outperformed other models and had higher accuracy levels. Furthermore, academic, demographic, internal assessment, and family/personal attributes were the most predominant input variables (e.g., predictive features) used for predicting student performance. Our analysis revealed an increasing number of research in this domain and a broad range of ML algorithms applied. At the same time, the extant body of evidence suggested that ML can be beneficial in identifying and improving various academic performance areas.

## 1. Introduction

Student academic performance is the most critical indication of educational advancement in any country. Essentially, students' academic achievement is influenced by gender, age, teaching staff, and students' learning. Predicting student academic success has gained a great deal of interest in education. In other words, student performance refers to the extent to which students achieve both immediate and long-term learning objectives [1]. Excellent academic record is an essential factor for a high-quality university based on its rankings. As a result, its ranking improves when an institution has a strong track record and academic achievements. From the student's perspective, maintaining outstanding academic performance increases the possibilities of securing employment, as excellent academic achievement is one of the primary aspects evaluated by employers [2].

The use of information technology (IT) in education can support institutions to achieve an improved educational outcome. For instance, in learning, artificial intelligence (AI) has a wide range of applications. AI-based technologies in education have grown in popularity to attract attention while improving quality and enhancing traditional teaching methods. For example, it facilitates gathering vast amounts of student data from multiple sources such as web-based education system (WBS) and intelligent tutorial system (ITS). Besides, these technological systems can provide data regarding students' grades, academic progress, online activities, and class attendance. Despite this, it is still challenging for educators to effectively apply these techniques to their specific academic problems due to the high volumes of data and rising complexity. As a result, it becomes difficult to accurately assess students' performance [3]. Therefore, the data obtained should be examined appropriately to identify factors that predict student success in the future.

Predicting and analyzing student performance are critical to assisting educators in recognizing students' weaknesses while helping them improve their grades. Likewise, students can improve their learning activities, and administrators can improve their operations [3, 4]. The timely prediction of student performance allows educators to identify low-performing individuals and intervene early in the learning process to apply the necessary interventions. ML is a novel approach with numerous applications that can make predictions on data [5]. ML techniques in educational data mining aim to model and detect meaningful hidden patterns and useable information from educational contexts [6]. Moreover, in the academic field, the ML approaches are applied to large datasets to represent a wide range of student characteristics as data points. These strategies can benefit various fields by achieving various goals, including extracting patterns, predicting behavior, or identifying trends [7], which allow educators to deliver the most effective methods for learning and to track and monitor the students' progress.

Our study was mainly motivated due to the lack of systematic and comprehensive surveys to assess the prediction of student academic performance using different ML models. Therefore, the main purpose of this work was to survey and summarize the key predictive features and the ML algorithms used to predict students' academic performance. The study's findings support mapping and assessing existing knowledge, research gaps, and future suggestions on further research carried out in this context.

The next section focuses on the methodology used in the systematic survey.  Section 2 provides a detailed summary of the results, while Section 4 discusses them. Lastly, the conclusion and future work are outlined in Section 5.

## 2. Methods and Materials

This work is conducted to assess the main ML algorithms and key attributes in student performance prediction. Several approaches [8–13] were followed, along with various strategies and steps proposed by references [10, 11] in performing this survey work. These include (a) formulation of research questions, (b) eligibility criteria, (c) information source/search strategy, and finally (d) study selection.

### 2.1. Research Questions

Forming the right research question is important to ascertain the key studies that are related to the prediction of student performance. Steps proposed in reference [13] were followed in order to formulate the right research questions (e.g., PICO framework), which represents the population, intervention, context, and outcome.  Table 1 summarizes the criteria of research questions.

Accordingly, this work is conducted to answer the following research questions:

- Q1: What are the key predictive features used in assessing the student performance?
- Q2: What are the key ML algorithms used in the prediction of student performance?
- Q3: What are the outcomes and accuracies of those ML algorithms?

### 2.2. Eligibility Criteria

We included studies that were (a) written in English, (b) published between 2015 and 2021, (c) from both conference proceedings and academic journals, (d) directly related to the prediction student performance focusing on ML, and (e) at any educational levels (Table 1). Furthermore, we excluded studies that were (a) not written in English, (b) in a form of traditional, conceptual, and systematic reviews, (c) other artificial intelligence (AI) methods such as deep learning (DL), and finally (d) not having empirical or experimental data.

### 2.3. Information Source and Search Strategy

A systematic and comprehensive search was performed to address the formulated research questions. For this objective, six online databases were searched in August 2021, including IEEE Xplore, ACM Digital Library, ScienceDirect, Scopus, Web of Science, and Google Scholar. A follow-up search was conducted at the beginning of October 2021 to identify any recently published works.

We used different terms of keywords, developed by Kitchenham et al. [14], and combined appropriately as follows: “prediction” OR “forecasting” OR “estimation” AND “student performance” OR “student academic performance” OR “academic achievement” OR “academic outcome” AND “machine learning” OR “ML” OR “data mining” OR “educational data mining.”

### 2.4. Study Selection

**Figure 1:**
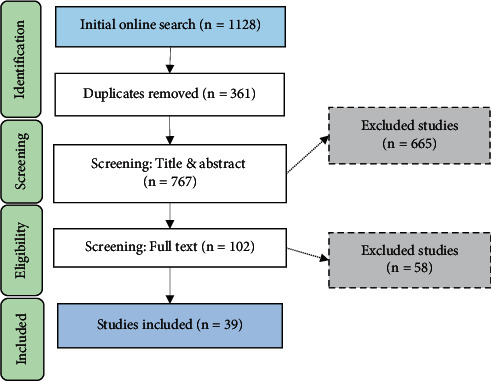
Articles screening and selection flowchart.

Two stages were performed for the screening and selection of the studies. Firstly, the selection of studies was based on the title and abstract screening, with regards to the eligibility criteria. Secondly, the selection of studies was based on a full-text assessment (see Figure 1).

We considered studies for full-text evaluation whenever there were any doubts. Disagreements between co-authors were reached by consensus. Furthermore, EndNote X20 software was utilized to remove duplicates and manage all citations.

Our search yielded 1128 papers. After eliminating duplicates, 767 papers remained. Six hundred of them were excluded based on title and abstract screening. The full text of the remaining 102 articles was considered and evaluated. Of these, 58 failed to meet the inclusion and exclusion criteria. The remaining thirty-nine relevant studies were evaluated for this review. Figure 1 illustrates the screening and selection procedures.

## 3. Results

### 3.1. Characteristics of the Included Studies

A total of twenty-six articles (66.7%) were published in academic journals, and thirteen articles (33.3%) were published in conference proceedings.

**Figure 2:**
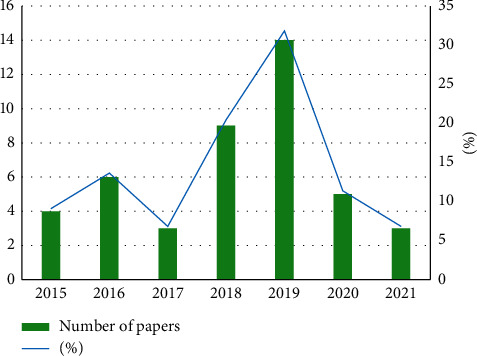
Number of publications per year.

The number of articles has significantly increased in recent years; this indicates that predicting students' performance through ML methods is attracting the attention of various scholars. As shown in Figure 2, most of the included articles were published between 2018 (*n* = 9, 23%) and 2019 (*n* = 14, 35%).

**Figure 3:**
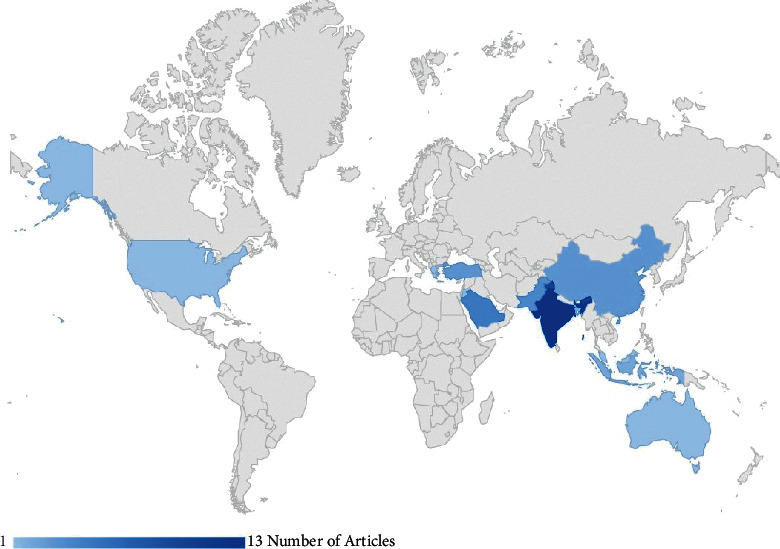
World map showing the distribution of studies per country.

According to the authors' affiliation countries, most published research was from India (*n* = 13, 33.3%), Saudi Arabia (*n* = 5, 12.8%), Pakistan (*n* = 4, 10.6%), and the other countries are between 1 and 2 articles (see Figure 3). Notably, over half of the studies (*n* = 36, 58%) on academic achievement in higher education analyzed data from an individual university.

**Figure 4:**
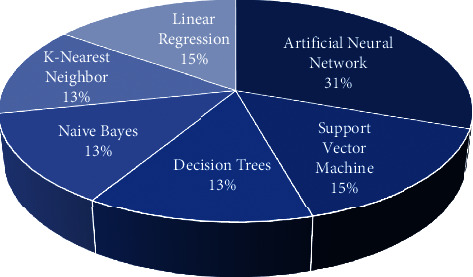
Distribution of machine learning approaches in student's performance.

Thirty-one percent (*n* = 14) of the ML methods used in predicting the student performance were artificial neural networks and support vector machine (*n* = 7, 15%). The remaining articles used decision tree, Naive Bayes, and K-nearest neighbor (*n* = 6, 13%).  Figure 4 represents the distribution of ML approaches used in the prediction. Regarding the classifiers used, most of the selected studies applied only one classifier and did not compare with others methods. Besides, six studies each tested four, three, and two classifiers. The highest number of classifiers used in studies wasten (*n* = 3). The majority of studies involving ANN mainly used one classifier.

Furthermore, the dataset applied in the studies ranged from 22 ([15]) to 20,000 ([16]). Especially, five studies ([17–21]) did not report the number of datasets used in their experiments. In most studies (*n* = 34), the datasets were divided and applied in both training and testing phases. However, five studies did not report the stages employed in their experiments.

### 3.2. Key Attributes Used in Predicting Student Performance

We grouped the attributes into seven categories: demographic, academic, internal assessment, communication, behavioral, psychological, and family/personal attributes (see Table 2). The most frequently used attributes were attendance and CGPA, which fall under the academic group. Twenty out of thirty articles have utilized the academic group to predict the performance of the students. This is because CGPA has significant academic potential.

The second most used attributes were gender, age, and nationality, which fall under the demographic group. Eighteen out of thirty-nine articles have used demographic attributes such as gender. The rationale behind thisis because male and female students have different learning styles [53]. Various studies have found that female students possess a more optimistic style of learning, positive attitudes, more discipline, and were self-motivated [54, 55]. Therefore, it is noticeable that gender has more significant influence on academic performance prediction.

Parent's status, survey, satisfaction, education, and income on the contrary, were the third most frequent attributes used in the prediction. These attributes fall under family/personal group, which has been used in eleven articles. Table 2shows the remaining attributes by category, name, and frequency.

### 3.3. ML Models Used in Predicting Student Performance

Accurate predictive modelling can be achieved by several techniques such as regression, classification, and clustering. However, we observed that classification is one of the most popular techniques used in predicting the academic performance. Several methods under a classifier have been used as listed in Table 3. Among these were artificial neural network (ANN), decision tree (DT), support vector machine (SVM), K-nearest neighbor (KNN), Naive Bayes (NB), and linear regression (LinR). The algorithms are highlighted in the subsections.

#### 3.3.1. Decision Tree (DT)

DT is often used due to its clarity and simplicity in discovering and predicting data. Many researchers noted that decision trees are easy to comprehend because they are built on IF-THEN rules [16, 61]. DT was used in six studies. The highest accuracy was 98.2% ([41]), while the lowest accuracy was 66% ([31]). The accuracy results of DT models are listed in Table 4.

#### 3.3.2. Linear Regression (LinR)

Linear regression defines the relationship of two variables through the data's adaptation of the regression line. As listed in Table 5, all seven articles had an average level of accuracy in predicting the student's performance. The highest accuracy level was 76.2% [51], and the lowest was 50% [48] in using LinR models.

#### 3.3.3. Artificial Neural Networks (ANNs)

The nonlinear and complex interaction between different input and output variables can be solved by using ANNs [62]. Our search yielded fourteen articles that used the ANN approach to predict the academic performance, as shown in Table 6. All ANN models in this work gave good results, with the maximum accuracy of 98.3% [18] and the lowest accuracy of 64.4%.

#### 3.3.4. Naive Bayes (NB)

Naive Bayes is highly scalable and requires several linear attributes to learn certain problems. We found six articles that applied the NB method in predicting the academic performance. The highest accuracy was 96.9% [49] and the lowest was 65.1% [42]).  Table 7shows the accuracy results of NB methods.

#### 3.3.5. K-Nearest Neighbor (KNN)

KNN stores and classifies classes based on a certain measure of similarity, such as distance function. As listed in Table 8, all six articles produced a high level of accuracy in predicting the student's performance. Notably, the highest accuracy was 95.8% [50], and the lowest was 69% [42].

#### 3.3.6. Support Vector Machine (SVM)

SVM is suitable for handling small datasets and has a greater generalization ability compared with other methods. Our search yielded seven articles that used the SVM approach. The maximum accuracy of the seven studies was 91.3% [40], and the lowest accuracy was 66% [20]. Futhermore, the accuracy of SVM is presented in Table 9.

**Figure 5:**
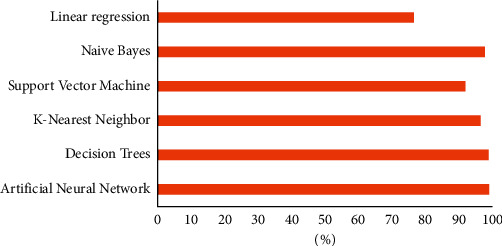
Prediction accuracy categorized by methods from 2015 to 2021.

Figure 5 illustrates the level of accuracy achieved by each approach in predicting student performance from 2015 to 2021. The maximum level of accuracy was achieved by using ANN models (98.3%).

The DTon the contrary, produced the second-highest accuracy (98.2%), followed by NB (97%) and KNN (95.8%). Furthermore, SVM, produced an accuracy of 91.3%. While, LinR had the lowest prediction accuracy compared to other methods (76%).

## 4. Discussions

This systematic survey focused on the existing ML techniques and critical variables used in predicting the academic performance of students, as well as the most accurate prediction algorithms. Table 3shows the prediction accuracy using classification methods grouped by algorithms for all selected studies from 2015 to 2021. Based on the data gathered in this work, supervised learning was the most extensively employed technique for predicting student performance, as it produces accurate and consistent findings. The ANN model, for instance, was the most widely applied by various scholars in fourteen studies and delivered the most reliable predictions. Furthermore, SVM, DT, LR, NB, and RF were well-studied algorithmic methods that produced good results. Similar to reference [64], unsupervised learning remains an unappealing approach for researchers, given their low accuracy in predicting students' performance in the current literature.

ANN demonstrated a remarkable accuracy (98.3%) in predicting student performance when combined with critical variables such as CGPA, gender, age, parent status, parent income, and family size. As a result, family status, parent's income, and family size can significantly affect student achievement. The DT is rated second with an average performance accuracy of 98.2%. GPA, grades, and demographics are the factors that led to the highest accuracy in predicting students' success in most of the studies that used DT. It can be concluded that DT can handle both forms of data and perform well in massive datasets, and the relationship between variables is simple to understand [65, 66].

NB has a performance accuracy of about 97%. According to these findings, demographic and academic characteristics are the best predictors of students' academic achievements, utilizing this approach. As a result, while using NB to predict student academic success, criteria such as gender, grades, results, and attendance should be addressed. The relevant variables included assignment course/subject and grades, while KNN had an average accuracy of 95%. The grade variable appears in ANN and DT as well. When applying Naive Bayes as a prediction method, the attributes used were significant. Furthermore, SVM has a performance accuracy of around 91%. From our analysis, the most appropriate attributes for predicting students' academic achievement using SVM are motivation, personality, learning tactics, and results. These criteria are considered significant in determining student academic success.

Finally, the method with the lowest prediction accuracy, with an average of 76%, was linear regression. Even though multiple factors were used in several studies, no significant variableswere identified. Gender, age, and final grades used in LinRstudies were also employed in KNN, DT, ANN, and NB. We presume that age and final grades were significant predictors of student performance.

To sum, prediction accuracy is determined by the traits or features employed throughout the prediction process [2]. As a result, we assume that ANN and DT approaches provided the best prediction accuracy due to the influence of primary qualities. According to earlier research [2], the CGPA factor increased accuracy in forecasting students' performance using the DT approach. Although the work of [15] has demonstrated that additional factors can influence a student's CGPA, more research is needed to identify the factors that substantially impact the CGPA. Academic features were the most commonly used variables, obtaining a score of 81% accuracy. It demonstrates that summative performance criteria such as CGPA, final grades, program, attendance, and topic are essential in forecasting student performance. This varies from a recent review by [64], revealing that GPA scores or ranges were employed less frequently in studies predicting student performance despite its importance.

## 5. Conclusions

Student performance prediction can assist educators in identifying student deficiencies towards improving their scores and enhancing learning. This study aimed to look at the latest ML algorithms and variables used to predict student academic performance. In our analysis, we identified 39studies from 2015 to 2021. Accordingly, the study findings showed a considerable rise of studies in this context recently. Furthermore, academics variables (e.g., CGPA and attendance), internal evaluations (e.g., quiz and assignment), demographics (e.g., gender), and family/personal characteristics significantly affect the prediction of students' performance.

Based on performance metrics, we conclude that the KNN classifier is an outstanding predictor of student achievement, followed by the DT technique. Predicting student academic achievement with high accuracy, on the other hand, demands a thorough grasp of the aspects and characteristics influencing student achievement. Given this, it is demonstrated that there are numerous potential areas for improvement in the design of the measurement devices used in instructor performance evaluation. Overall, this is still a developing subject, and future studies are expected to include more algorithms for greater accuracy.

Our analysis suggests that first, a new set of inputs and a more robust and extensive dataset are necessary for greater accuracy. Second, it is suggested that data to be gathered from multiple institutions to combine the environment-dependent qualities are not addressed in the extant literature. Third, for a more efficient classification technique, improving the ideal selection of qualities is necessary based on their connection. Finally, to thoroughly assess a model's performance, precision and recall need to be measured.

## Figures and Tables

**Table 1:** PICO framework for developing research questions.

PICO criteria	Description
Population	Male/female students; above 17 years; all educational levels.
Intervention	Machine learning (ML) algorithms.
Context	Academic institutions; university; college; high school.
Outcome	Model accuracy; key predictive features and models.

**Table 2:** Attributes used in the prediction of student's performance.

Attribute category	Attributes	Frequency	Study reference
Demographic	Gender; age; nationality; place of birth; marital status; guardian; address; transport	21	[3, 17, 19, 20, 22–38]
Academic	CGPA; stage ID; grade ID; section ID; topic; semester; program; attendance; final grade	20	[15, 17, 19, 20, 22–27, 30–32, 34, 37, 39–41, 41, 42], References [36–38, 43–45].
Internal assessment	Coursework; assignments; quizzes; lab test; midterms; examinations; daily study time; plagiarism counts; virtual learning access; group presentation; personal report	15	[3, 15, 18, 19, 21, 36, 37, 39, 40, 42, 43, 46–49]
Family/personal	Parent status; parent survey; parent satisfaction; family size; parent education; parent job; income; travel time; Study time; free time; health	12	[3, 20, 22, 23, 26, 28, 33–37, 39, 50]
Behavioral	Raised hands; visited resources; announcement view; discussion	5	[3, 20, 22, 26, 34, 51]
Communication	Messages; emails; response time; login/Logout time; time spent; number of words; voting system	4	[18, 25, 43, 46]
Psychological	Personality; motivation; contextual influences; learning strategies; socio economic status; approach to learning	2	[40, 52]

**Table 3:** Main classifiers used in the selected studies.

Algorithm	Average accuracy (%)	Study
Artificial neural network (ANN)	85.9	[17, 18, 22–25, 25, 26, 36, 39, 46, 56–58]
Decision tree (DT)	85	[29–31, 36, 41, 59]
Support vector machine (SVM)	83.4	[1, 16, 20, 27, 28, 40, 52]
K-nearest neighbor (KNN)	80.7	[32–35, 43, 50]
Naive Bayes (NB)	83	[3, 15, 19, 42, 49, 60]
Linear regression (LinR)	55.5	[37, 38, 44, 45, 47, 48, 51]

**Table 4:** Accuracy results for decision tree (DT).

Study	Year	Predictive features	Accuracy (%)
[41]	2016	Student ID, graduation GPA, high school score, general aptitude test (GAT), educational attainment test (EAT), and courses	80
[59]	2019	Final examination, continuous assessment, schooling marks, quizzes, assignments, class test, and midterm examinations	98.2
[29]	2019	Gender, school name, travel time, age, hobbies, health details, and address	97.9
[30]	2019	Student demographics, student grades, subjects, school-related information, and social activities	95.8
[31]	2019	Gender, age, family size, health, marital status, work status, school grade, university type, faculty type, scholarship, transportation, traveling time, credit hours, study time, and GPA	66
[36]	2020	Gender, age, address location, parent job, Travel time, study time, free time, failures, activities, health, and abstance	72.26

**Table 5:** Accuracy results for linear regression (LinR).

Study	Year	Predictive features	Results
[51]	2015	Total playing time, number of videos played, number of rewinds, number of pauses, number of fast forwards, and number of slow play rate use	Accuracy = 76.2%
[44]	2016	Course-specific subdata	RMSE = (0.63, 0.72), Precisition = 26.86%.
[47]	2018	Exercises, homeworks, and quizzes	pMSE = 198.68, pMAPC = 0.81
[48]	2018	Number of views/post of student, course information, student information, submitted assignments, and progress of assignments	Accuracy = 50%
[45]	2018	Summative evaluation attributes	Accuracy = 69%
[37]	2020	Gender, age, parent education, family size, test preparation, father job, mother job, absent days, parent status, travel time, and academic scores	—
[38]	2020	Final grades	—

**Table 6:** Accuracy results for artificial neural networks (ANNs).

Study	Year	Predictive features	Accuracy
[17]	2015	Gender, location, type of school, high school score, CGPA, number of credits, and results	84.6%
[39]	2016	Test mark, class and lab performance, attendance, assignment, study time, previous result, family education, living area, drug addiction, affair, social media, and final year results	88%
[18]	2016	Online quizzes, email communication, content creation, and content interaction	98.3%
[22]	2018	Grades, gender, nationality, place of birth, section ID, topic, raised hand, discussion, class in 1st and 2nd terms, attendance, and parent satisfaction	85.4%,
[23]	2018	Gender, attendance, results, economic status, and parental education	-
[24]	2019	Gender, CGPA, English, Chinese, math, science, and proficiency test	84.8%
[25]	2019	Gender, content score, time spent, homework score, and attendance	80.5%
[46]	2019	CourseID, total of learning sessions, length of sessions, total of assessments of semester 1, grades, quizzes, and emails sent	97.4%
[26]	2019	Gender, nationality, place of birth, StageID, GradeID, SectionID, topic, semester, relation, raised hands, discussion, parent survey and satisfaction, and attendance	73.5%
[36]	2020	Gender, age, address location, parent job, travel time, study time, free time, failures, activities, health, and abstance	64.40%
[56]	2021	Gender, region, educational level, age range, neighborhood crime rate (IMD), number of times they have previously participated in the course, enrolled credits, disability, and the final exam result (passed/failed). In addition, the number of times the student has interacted with any of the online course contents has been counted throughout the courses	78.20%
[63]	2020	Gender, content score, time spent, number of entries to content, homework score, attendance, and archived courses	80.47%
[57]	2021	123 variables	82.10% (high) 70.89% (low)
[58]	2021	116 features for the production and 84 for the learning phase	80.76% and 86.57%

**Table 7:** Accuracy results for Naive Bayes.

Study	Year	Predictive features	Accuracy (%)
[42]	2015	Attendance, internal grade, computer skills, school level, mobile, tuition, type of school, type of board, and gender	65.1
[3]	2016	Age, section, program, method, place of birth, transport, subject, motivation level, homework, tuition, parent education, attendance, communication, GPA, quiz, assignment, lab test, and final exam	86
[60]	2017	List of subjects and grades	83.6
[19]	2018	Gender, age, admission, attendance, study mode, program, education status, book resources, and quiz	72.4
[15]	2018	CGPA, high risk, coursework, examination, plagiarism count, campus access, and off-campus access	90
[49]	2015	Number of views/post of student, course information, student information, submitted assignments, and progress of assignments	96.9

**Table 8:** Accuracy results for K-nearest neighbor.

Study	Year	Predictive features	Accuracy (%)
[32]	2017	Gender, age, knowledge score, skill score, CGPA, group heterogeneity, and label class	95.5
[33]	2017	School, gender, address, family size, parent status, parent job, guardian, support, activities, nursery, internet, and romantic relationship	93
[50]	2018	Parent income, semester, family members, and CGPA	95.8
[34]	2019	Nationality, gender, place of birth, parent responsibility, stages, grades, SectionID, topic, attendance, semester, raised hand, visited resource, discussion, and parent satisfaction	69
[35]	2019	Gender, age, school, address, parent status, parent education, parent job, family size, guardian, travel time, and study time	88
[43]	2020	Absence, virtual learning access, voting system result, presentation result, and personal report result	74

**Table 9:** Accuracy results for support vector machine (SVM).

Study	Year	Predictive features	Accuracy (%)
[40]	2016	Attendance, class time, class length, instructor knowledge, instructor appearance, performance, assignments, exams, course materials, communication, motivation, learning outcomes, and grades	91.3
[16]	2018	Specialization, subject, programming skills, analytical skills, personal details, memory, workshops, certifications, and sports	90.3
[27]	2019	Gender, race, grades, and subjects	77
[20]	2019	Gender, nationality, place of birth, relation, StageID, SectionID, GradeID, topic, semester, raised hands, visited resources, announcement view, discussion, parent satisfaction, and attendance	66
[52]	2019	Motivation, personality, learning strategies, socio-economic status, learning approach, and psychosocial influences	90
[28]	2019	Performance, subjects, parental status, family size, location, and address	79.4
[36]	2020	Gender, age, address location, parent job, Travel time, study time, free time, failures, activities, health, and abstance	71.2

## Data Availability

The data supporting this review are from previously reported studies and datasets, which have been cited. The processed data are available from the corresponding author upon request.
